# Morphological differences in tardigrade spermatozoa induce variation in gamete motility

**DOI:** 10.1186/s40850-022-00109-w

**Published:** 2022-01-30

**Authors:** Kenta Sugiura, Kogiku Shiba, Kazuo Inaba, Midori Matsumoto

**Affiliations:** 1 Department of Biosciences and Informatics, Faculty of Science and Technology, Keio University, 3-14-1 Hiyoshi, Kohoku, Yokohama, Kanagawa 223-8522 Japan; 2 Shimoda Marine Research Center, University of Tsukuba, 5-10-1 Shimoda, Shizuoka, 415-0025 Japan

**Keywords:** Tardigrade, Mating, Spermatozoa, Motility, Fertilization, Reproduction, Paramacrobiotus sp, Macrobiotus shonaicus, Imaging, Morphology

## Abstract

**Background:**

Fertilization is an event at the beginning of ontogeny. Successful fertilization depends on strategies for uniting female and male gametes that developed throughout evolutionary history. In some species of tardigrades, investigations of reproduction have revealed that released spermatozoa swim in the water to reach a female, after which the gametes are stored in her body. The morphology of the spermatozoa includes a coiled nucleus and a species-specific-length acrosome. Although the mating behaviour and morphology of tardigrades have been reported, the motility of male gametes remains unknown. Here, using a high-speed camera, we recorded the spermatozoon motilities of two tardigrades, *Paramacrobiotus* sp. and *Macrobiotus shonaicus,* which have longer and shorter spermatozoa, respectively.

**Results:**

The movement of spermatozoa was faster in *Paramacrobiotus* sp. than in *M. shonaicus*, but the beat frequencies of the tails were equal, suggesting that the long tail improved acceleration. In both species, the head part consisting of a coiled nucleus and an acrosome did not swing, in contrast to the tail. The head part of *Paramacrobiotus* sp. spermatozoa swung harder during turning; in contrast, the tail of *M. shonaicus* moved more widely than the head. Finally, after mating, the spermatozoa that reached the female aggregated around the cloaca while waiting to enter her body in both tested species.

**Conclusions:**

This study provides results for the first observations and analyses of individual spermatozoon motility in tardigrades. A comparison of the spermatozoon movements of the two tardigrades suggested that the motilities of the male gametes were affected by morphological differences, where the longer spermatozoa swam faster and the shorter ones showed more stable swimming. Swimming was mainly induced by tail movement, but the long head of *Paramacrobiotus* sp. spermatozoa might be especially important for turning. In addition, observations of mated female cloacae suggested that the head parts of the spermatozoa were required for aggregation around the cloaca of a mated female.

**Supplementary Information:**

The online version contains supplementary material available at 10.1186/s40850-022-00109-w.

## Background

**Fig. 1:**
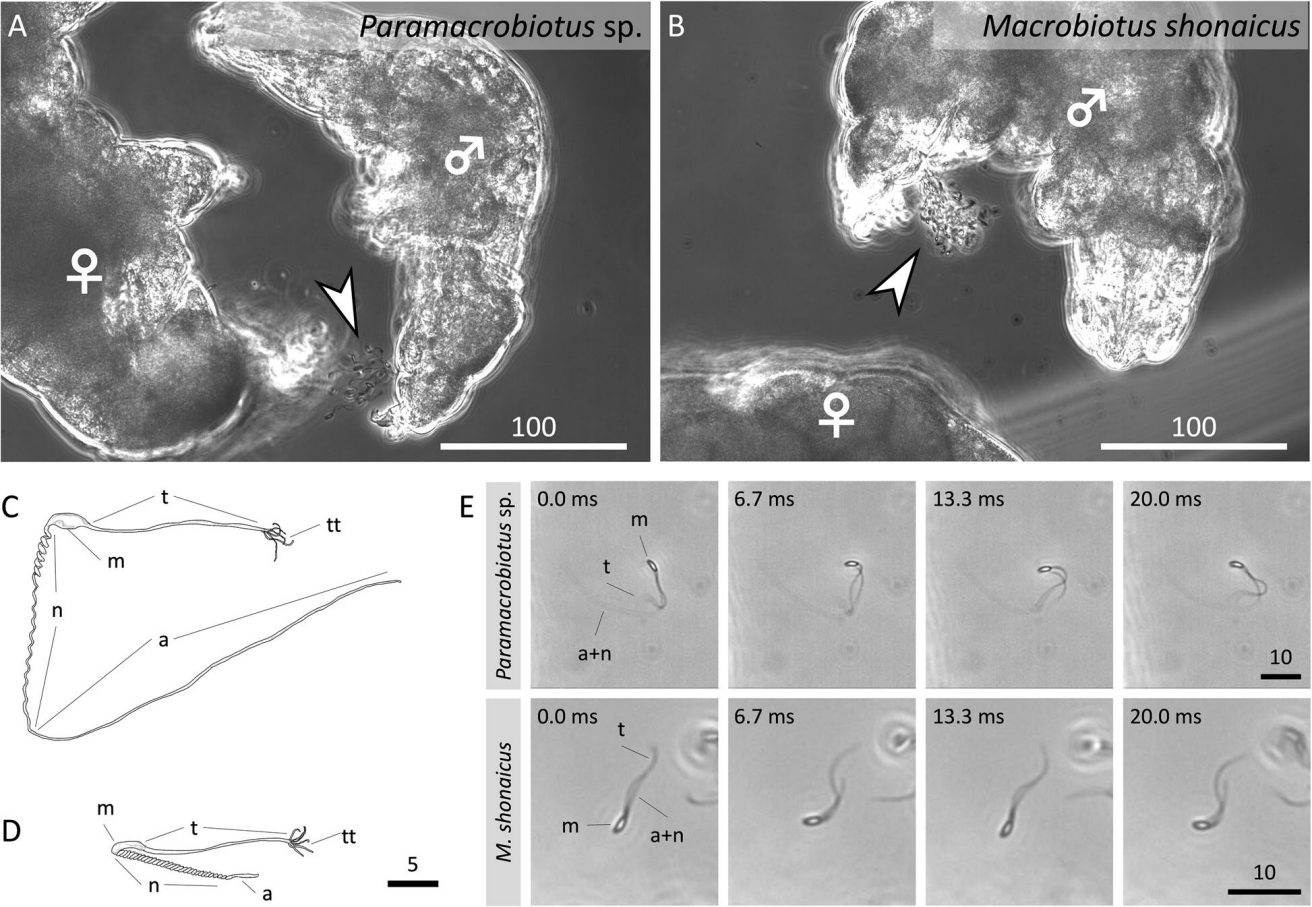
Observations of the ejaculated spermatozoa **A**&**B** The ejaculated spermatozoa were recorded. Arrow heads indicate the released spermatozoa. **C**, **D** Schemas of spermatozoa of *Paramacrobiotus* sp. and *M. shonaicus*, respectively. **E** Time lapse images of spermatozoa swimming. Scale bars: μm. Abbreviations a, m, n, t and tt indicate acrosome, mid-piece, nucleus, tail and tuft, respectively

Fertilization is an important event in the production of the next generation in amphimictic animals. Female and male gametes meet, and then ontogeny begins. Modes of fertilization are generally divided into two types: internal and external fertilization. In the internal mode, the union of gametes occurs inside the female’s body. In contrast, in the external mode, fertilization begins in the environment. In both cases, the behavioural strategies of spermatozoa for reaching eggs show diversity and are required to increase the rate of successful fertilization [1]. Tardigrades, often called water bears, are aquatic animals in which females lay their eggs in their moulted exuvia or directly into the environment [2]. In relation to the former scenario, fertilization is thought to occur externally in *Isohypsibius dastychi* (Pilato, Bertolani & Binda, 1982) because male ejaculation and female oviposition are simultaneous processes [3, 4], but in *Pseudobiotus megalonyx* (Thulin, 1928) and *Ursulinius nodosus* (Marray, 1907), internal fertilization is presumed because ejaculation occurs before egg deposition, with spermatozoa enter in the oviduct via cloaca opening [5–9]. On the other hand, in species that have a spermatheca and lay their eggs freely, internal fertilization is expected because there is a spermatheca connected to the oviduct between the cloaca and ovary [9–12]. However, observations of egg laying in *Paramacrobiotus* sp. and *Macrobiotus shonaicus* (Stec, Arakawa and Michalczyk, 2018) indicated that fertilization begins after eggs are laid into the environment [13, 14]. Moreover, observations of mating behaviours have revealed that the spermatozoa of the two species are first ejaculated into the environment, after which they swim to the mating female and are stored in her spermatheca; then, oviposition occurs [13, 15]. The morphologies of the spermatozoa have been well reported not only in these two species but also in other species belonging to the same family, Macrobiotidae, and demonstrated that the spermatozoa have a tail, an inflated or rod-shaped mid-piece, a coiled nucleus and an acrosome [10, 14, 16–20] (Fig. 1C, D). Morphological analyses and comparisons have been performed, indicating that the length of the acrosome is extremely variable among genera and suggesting that the functions of the spermatozoa are affected by morphological characters. However, information on the motility of tardigrade spermatozoa is lacking.

Here, we observed the spermatozoon motilities of two tardigrades, *Paramacrobiotus* sp. and *M. shonaicus*, which are the species the mating behaviours and spermatozoon morphologies have been confirmed [13, 14]. According to morphometric research, the spermatozoa of *Paramacrobiotus* sp. and *M. shonaicus* had longer and shorter acrosomes, respectively; thus, we examined and compared their movement as affected by morphological variation.

## Results

### Observation and tracking of ejaculated spermatozoa

Ejaculated tardigrade spermatozoa in a droplet within the 0.5 μm gap under cover slips were observed under an inverted-phased microscope with a stroboscopic flash and a high-speed camera (Fig. 1A, B). The recorded movies demonstrated the detailed motilities of the spermatozoa. Each spermatozoon included a head part (an acrosome and a nucleus) and a tail (Movies S1, S2, Fig. 1C-E). The gametes swam by leading with the mid-piece (Movie S3).

**Fig. 2:**
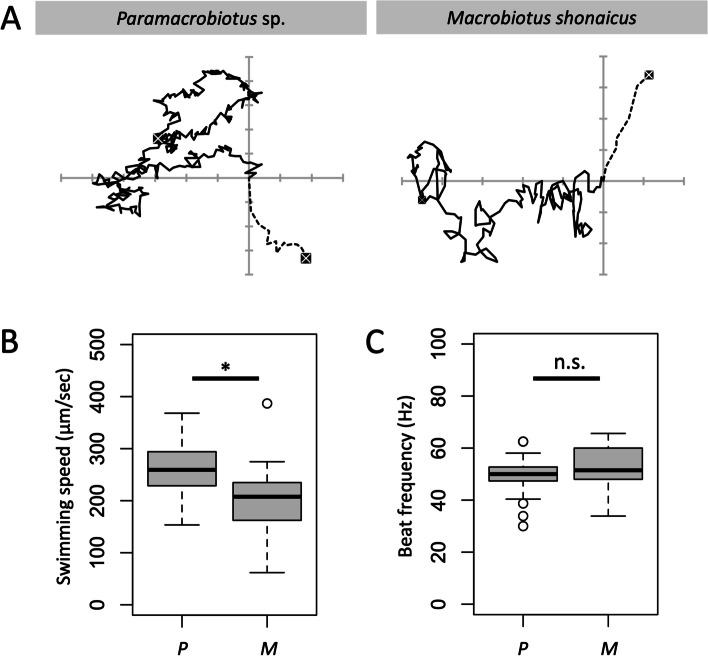
Tracks, speeds and beats of the spermatozoa **A** The longest (black lines) and shortest (dotted lines) swimming tracks of the respective species. "x" indicates the start position. **B** The swimming speeds of the spermatozoa. **C** The beat frequency of tails. *: *p* < 0.01 in the Mann–Whitney U-test, n.s.: no significant difference

Most of the released spermatozoa were able to reach a female during the observation period, but some could not. The trajectories of the spermatozoa that reached a female were straight and/or curved (Fig. 2A, Movie S3). The swimming speeds were 259.3 μm/sec and 207.6 μm/sec in *Paramacrobiotus* sp. and *M. shonaicus,* respectively (Fig. 2B), and the spermatozoa of the former moved significantly faster (*n* > 25, Mann–Whitney U-test, *p* < 0.01). The beat frequency of the tails during swimming was 49.5–52.5 Hz on average (Fig. 2C) and did not differ significantly between species (*n* > 26, Mann–Whitney U-test, *p* < 0.1).

### Obtaining coordinates of the motilities

**Fig. 3:**
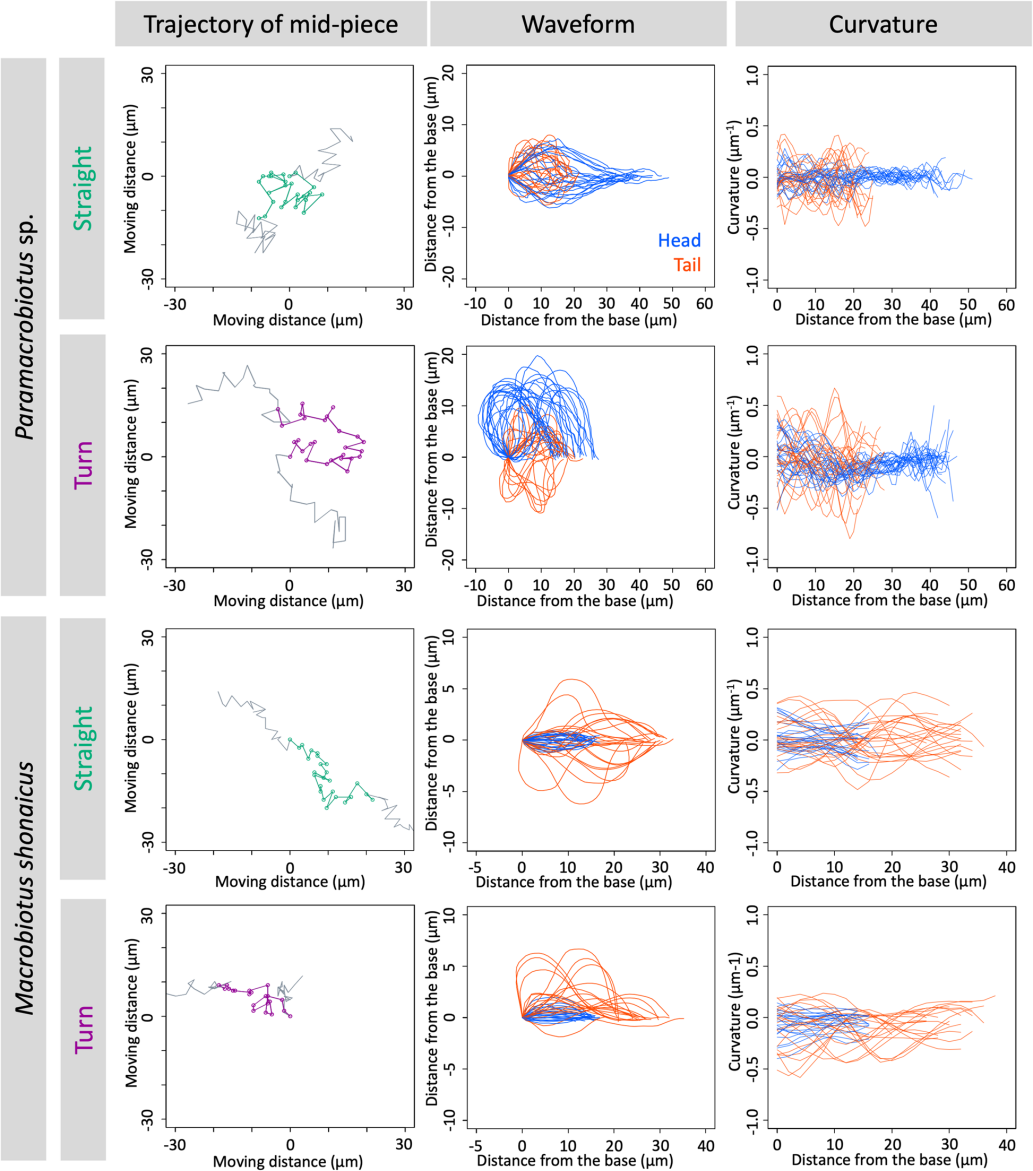
Spermatozoa trajectories Obtained trajectories, normalized waveforms and curvatures of the heads and tails in the respective species. “Straight” and “Turn” indicate a spermatozoon swimming in a straight line and turning, respectively. Coloured lines of obtained trajectories correlate to waveforms in right panels

The motilities of the head parts and tail parts were analysed using Bohboh software (Bohboh Soft, Tokyo, Japan) (Movie S3). From the obtained coordinates, trajectories of the mid-piece, head and tail were visualized (Fig. 3). In addition, normalized waveforms and curvatures corresponding to the length were calculated (Fig. 3, Movie S3).

**Fig. 4:**
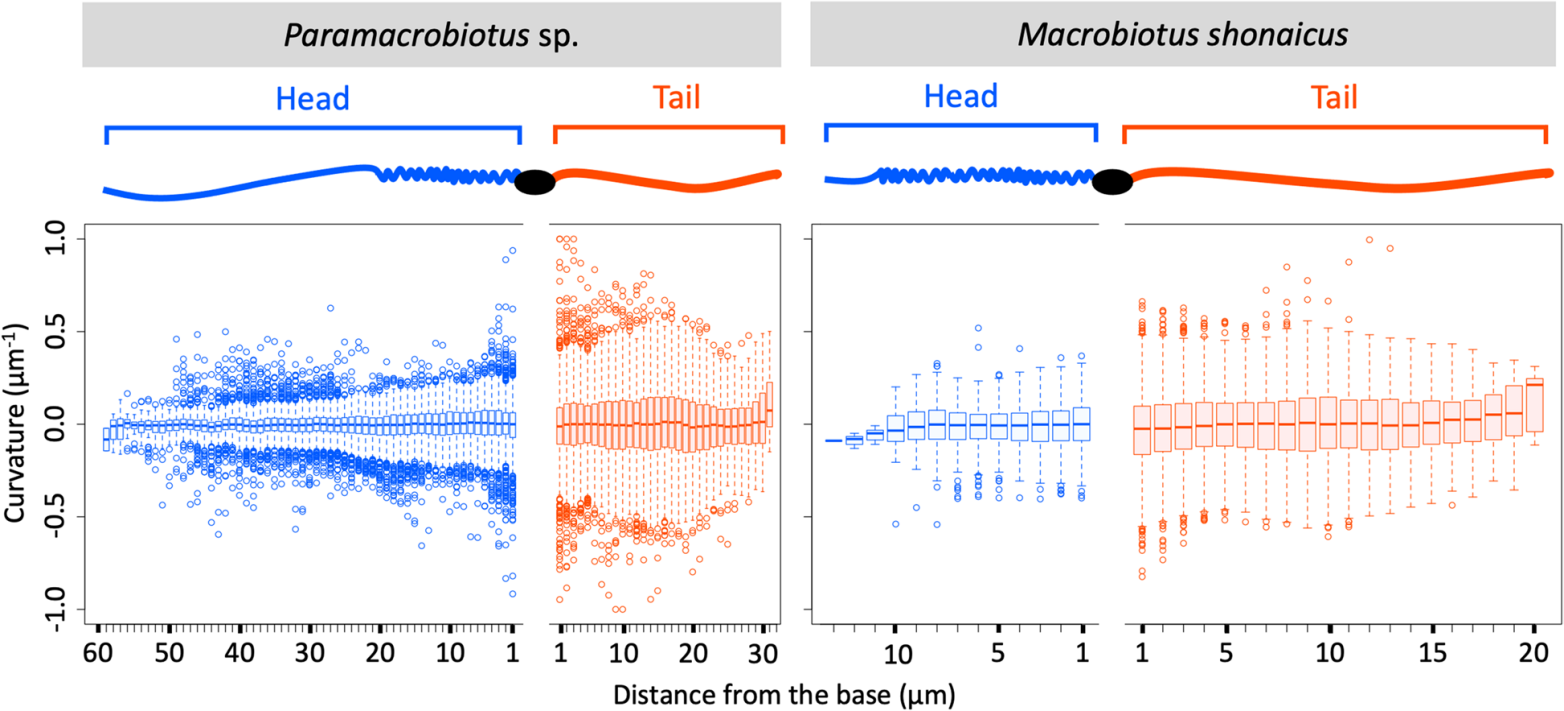
Spermatozoon motility comparison between parts and species The curvatures of spermatozoa of *Paramacrobiotus* sp. (left) and *M. shonaicus* (right)

To compare the amplitude between the head and the tail, the transition of the curvature correlating to the distance from the jointing point was obtained. In the comparison, the head parts trembled less than the tail parts (Fig. 4, F-test, *p* < 0.01). In addition, the obtained data showed differences in amplitude between the tested species. The head curvatures of the two species showed significant differences in 63.6% of the compared positions (1–3, 6–7, and 9–10 μm from the base, F-test, *p* < 0.01). In the comparison of the tails, the curvatures were different in the 12–16 μm position (26.3% of compared positions, F-test, *p* < 0.01).

### Comparison of motility curvatures

**Fig. 5:**
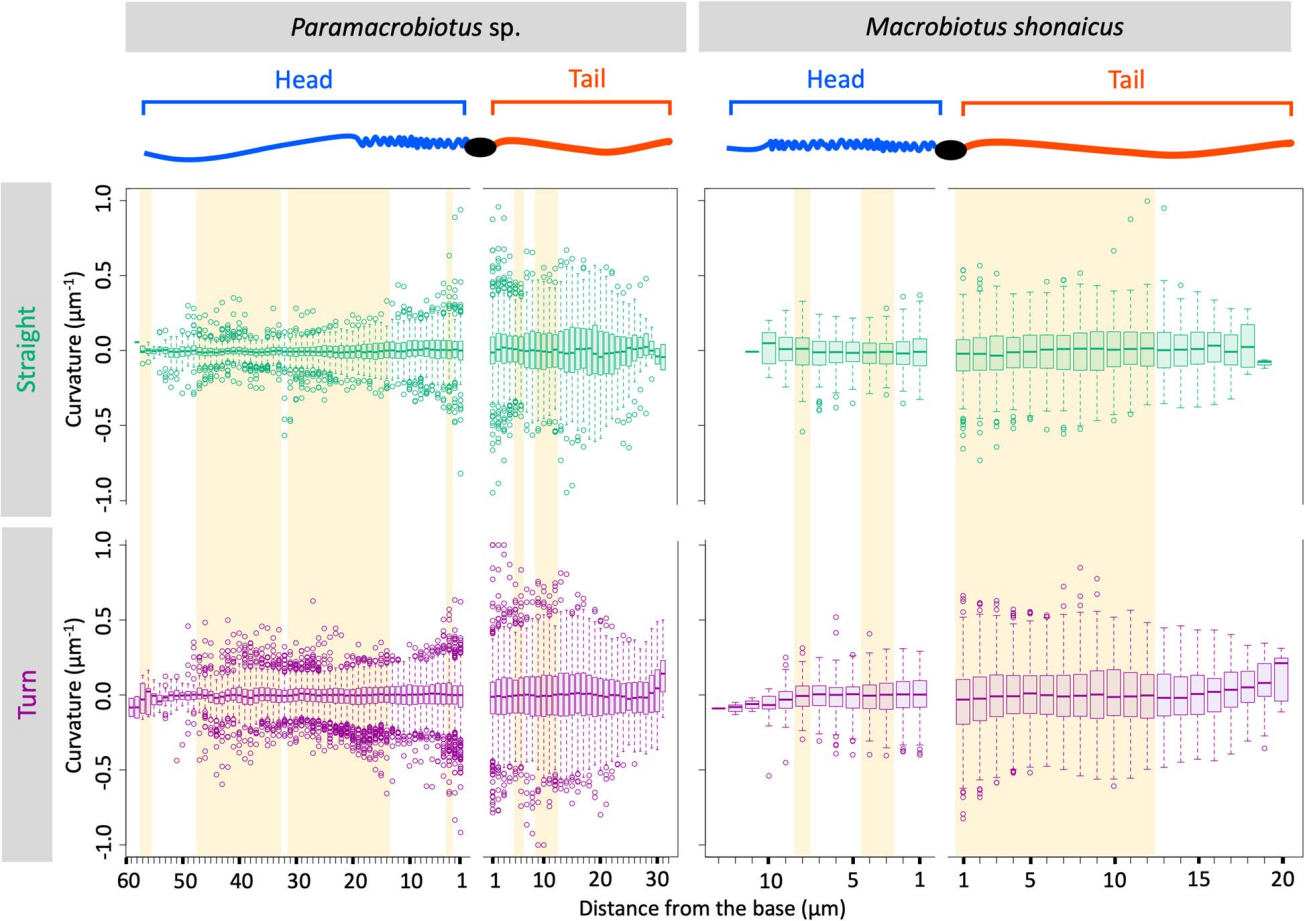
Spermatozoon movement comparison between swimming in a straight line and turning Green and purple boxplots represent the curvatures of spermatozoa swimming in a straight line and turning, respectively. Yellow highlighted positions show significant differences in their movements (F-test, *p* < 0.01)

The obtained curvatures were categorized according to the direction of swimming (in a straight line or turning), and a comparison of motility curvatures in the two swimming patterns showed intraspecies differences in movement. In *Paramacrobiotus* sp., 61.0% (36 positions/59 positions in total) of the head regions were significantly curved during turning compared with swimming in a straight line; in addition, the positions were concentrated in the range 13–48 μm (Fig. 5). The tail parts were more curved in 6 of the 31 positions (19.4%). In contrast to the results in *Paramacrobiotus* sp., 3/13 positions (23.1%) of the head parts and 12 out of 21 positions in total (57.1%) of the tail parts were widely curved during turning in *M. shonaicus* (Fig. 5).

### Spermatozoa around the female’s cloaca

**Fig. 6:**
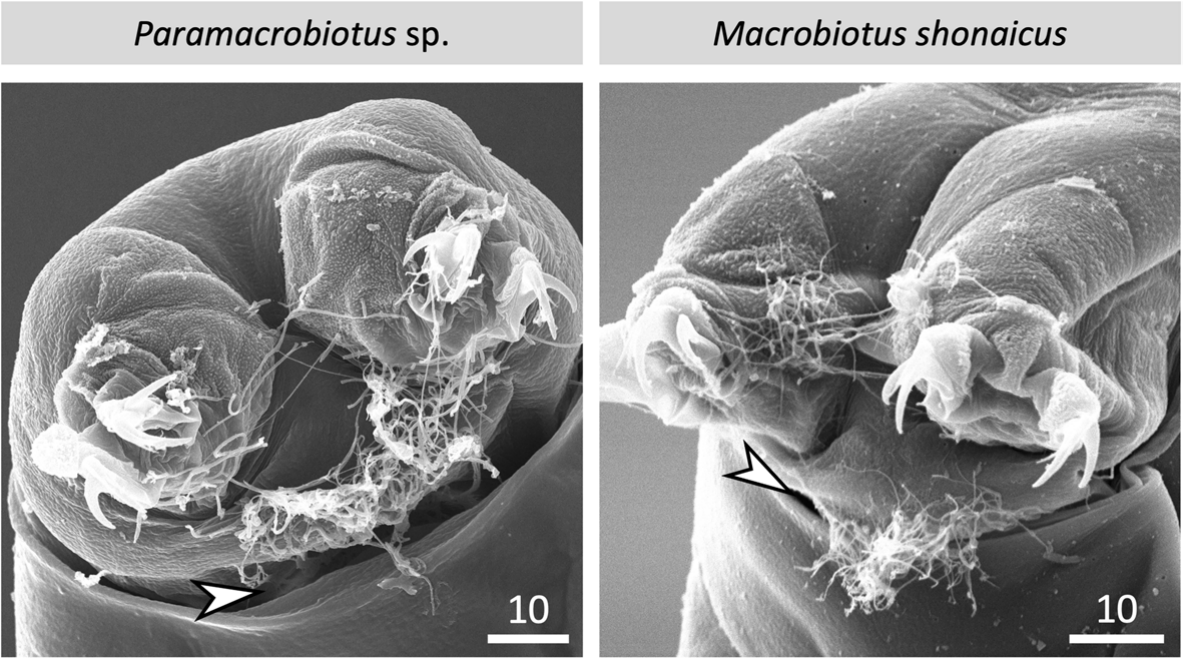
Spermatozoon aggregation SEM photos of the cloaca of a mated female. The claws of legs IV are visible. Arrow heads indicate the cloaca. Scale bars: μm

Fifteen minutes after ejaculation, the cloacae of the mated females were observed with scanning electron microscopy (SEM). The observations revealed that numerous spermatozoa aggregated around the cloaca and the leg IV (Fig. 6).

## Discussion

In this study, we performed the first detailed observations of the motilities of tardigrade spermatozoa. The observations clearly revealed that the spermatozoa swam with the mid-piece in the front position and moved in a straight line and/or turned. Additionally, the swimming speeds in two species were measured. The spermatozoa of *Paramacrobiotus* sp. were faster than those of *M. shonaicus*. The spermatozoa of the genus *Paramacrobiotus* were generally longer than those of the genus *Macrobiotus*; moreover, the extraordinary head length of spermatozoa in the genus *Paramacrobiotus* was considered to enhance spermatozoon movements [19]. Our results support this hypothesis.

The curvature values of the tails were significantly larger than those of the heads in both species. The results indicated that the movements were mainly induced by the tails. In other aquatic animals, for example, ascidians and sea urchins, although the head parts are not as long as those in tardigrades, the swimming of the spermatozoa is induced by tail movement [21]. The tails, called flagella, are formed by axonemes, and the configuration is widely conserved [22]. Axonemes have been observed in the spermatozoa of some tardigrade species; in addition, the constructions were similar to those in other animals [10, 19, 23–27]. Unfortunately, ultrastructural information for the tested species *Paramacrobiotus* sp. and *M. shonaicus* is lacking, but the formation of the axoneme is also thought to be conserved in the species, suggesting that tail motility is induced by the same mechanism. In addition, the motilities of each part were significantly greater in *Paramacrobiotus* sp. than in *M. shonaicus*, suggesting that the longer heads and tails of *Paramacrobiotus* sp. spermatozoa enhanced their movement [14].

In a comparison of the trajectories of the spermatozoa, the tails of the turning spermatozoa in *M. shonaicus* curved more dramatically than when the spermatozoa were swimming in a straight line, indicating that the determination of direction depended on the tail's movements. On the other hand, the extraordinarily long head parts of *Paramacrobiotus* sp. spermatozoa showed increased curvature during turning, suggesting that the direction, i.e., whether turning or swimming in a straight line, was affected by longer parts than the tail. According to our results, the acrosome is more flexible than the coiled nucleus because the tip part of *Paramacrobiotus* sp. spermatozoa swung widely. In contrast, the flexible component of *M. shonaicus* spermatozoa is short; therefore, the head parts did not curve as much as those in *Paramacrobiotus* sp., allowing more stable swimming. Most spermatozoa reached the female’s cloaca in a few seconds. However, the spermatozoa remained aggregated around the cloaca for 15 min after mating, suggesting that they waited to enter the spermatheca of the female.

The correlation of spermatozoon and egg morphologies in Macrobiotidae was suggested [26]; for example, eggs of species that have longer acrosomes are generally conical. On the other hand, eggs of species with shorter acrosomes are usually ornamented and often have an inverted-goblet shape. Generally, the shapes of gametes should have some functional significance for efficient and certain reproduction. A previous study hinted at the existence of gamete morphological interference at the phase of fertilization in some species [14]. Although this study focused only on male gametes, the comparisons of motility in relation to length might provide insight into the morphological differentiation that affects one of the functions of spermatozoa. To reveal the factors affecting and benefits of morphological variation in tardigrade spermatozoa, we believe it is important to accumulate observations and knowledge in many species. As a related species, *Mesobiotus* spp. could support next studies because the morphology of its spermatozoon was intermediate between the genus *Paramacrobiotus* and *Macrobiotus* [28]. In future investigations, if similar knowledge is accumulated for various species as is done in this study, evolutionary history can be inferred based on species differentiation in gamete features.

## Conclusions

In summary, we performed detailed observations of swimming spermatozoa of tardigrades. A comparison of movements between spermatozoa with longer versus shorter acrosomes demonstrated differences in swimming speeds, waveforms and curvatures, indicating that the morphological differences of the spermatozoa in tardigrades influence their movements. The waveform differences between swimming directions suggested that the long head of *Paramacrobiotus* sp. spermatozoa affects turning. In addition, SEM observations of mated female cloacae indicated that the released male gametes aggregate around the cloaca at least 15 min after mating.

## Methods

### Tardigrade culture conditions and specimen preparation

Two tardigrades, namely, *Paramacrobiotus* sp. TYO strain and *M. shonaicus*, were used for this investigation. These tardigrades were provided by Dr. Takekazu Kunieda and Dr. Kazuharu Arakawa, respectively. The culture conditions were the same as those described in Sugiura et al. [13]. The tardigrades were placed in plastic dishes containing 1.2% agar gel (Nacalai Tesque, Kyoto, Japan) topped with mineral water (Volvic) and then kept in the dark at 20℃. The rotifer *Lecane inermis* and the green alga *Chlorella vulgaris* (Recenttec KK, Tokyo, Japan) were added to the culture as food sources. The water was changed every 3–5 days, and the dishes were renewed every 2–3 weeks. Sample preparations for mating observations also followed Sugiura et al. [13]. Females in stage 3–4 that showed tightly packed ovary were isolated in a separate culture for at least one week, and males with testes were separated into a different culture to prevent unexpected mating. Observations until this step were performed under a stereomicroscope (SZX10, Olympus, Tokyo, Japan).

### Recording motilities of the spermatozoa

A pair of tardigrades, a female and a male, was selected to observe mating. The specimens were suspended in a droplet of the mineral water placed within a 0.5 μm gap created by a U-shaped silicon sheet (AS ONE, Osaka, Japan) between 18 mm × 18 mm and 50 mm × 24 mm cover slips (Matsunami Glass Ind., Ltd., Osaka, Japan). The behaviours of the tardigrades were observed under an inverted-phased microscope (IX71, Olympus, Tokyo, Japan) with a 20 × objective lens. The field was illuminated with a laboratory-made red stroboscopic LED lamp (620–630 nm, Power LED, Edison, Taiwan). The swimming motility of the released spermatozoa was video recorded using a high-speed charge-coupled device (CCD) camera (HAS-U2, DITECT, Tokyo, Japan) at 150 frames per second (fps) [29].

### Analyses of spermatozoon motility

The coordinates of the spermatozoon was obtained by tracking the mid-piece as an indicator in ImageJ software [30], and the speed of movement (velocity curvilinear: VCL [29]) was calculated from the moving distance and the frames of the video on R software [31]. Computer-assisted sperm analysis (CASA) was mainly performed with Bohboh software (Bohboh Soft, Tokyo, Japan) [29]. The spermatozoa were divided to a head part (including a nucleus and an acrosome) and a tail part, and the coordinates of the two regions were obtained from the base (junction with the mid-piece) to tip of each part in each one micrometre position over 10 sequential frames. Normalized waveforms and curvatures were determined with the software and visualized with ImageJ or R [30, 31]. The beat frequency was calculated by counting the wave peaks in the 5 μm position of the tails and dividing the count by the elapsed time. Motility was categorized as swimming in a straight line or turning. Distinction of the nucleus and acrosome was performed according to morphometrical data available in Sugiura and Matsumoto [14] because of limited resolution. Specifically, the nucleus was defined as 0–21 μm and 0–11 μm in *Paramacrobiotus* sp. and *M. shonaicus*, respectively, and the acrosome was defined as the remaining region.

### Statistical comparisons

Statistical comparisons were performed with a Mann–Whitney U-test or an F-test in R [31], using the “wilcox.exact” and “var.test” functions, respectively.

### SEM observations

SEM observations were performed as described in Sugiura and Matsumoto [14]. Fifteen minutes after mating, eight females of each species were dehydrated in 100% ethanol for 3 h and then soaked in tertial-butyl alcohol overnight. After being lyophilized with a JFD-320 device (JEOL, Tokyo, Japan), the samples were transferred onto aluminium stubs. The samples were then sputter-coated with gold and observed with a JSM 6510 (JEOL) scanning electron microscope.

## Supplementary Information

**Additional file 1.** 

**Additional file 2.** 

**Additional file 3.** 

**Additional file 4.** 

## Data Availability

The datasets used and/or analyzed during the current study are available from the corresponding author.

## Abbreviations

CASA: Computer-assisted sperm analysis
CCD: Charge-coupled device
FPS: Frames per second
SEM: Scanning electron microscopy
VCL: Velocity curvilinear
